# Influence of carbon emission trading policy on residents' health in China

**DOI:** 10.3389/fpubh.2022.1003192

**Published:** 2022-09-21

**Authors:** Bingnan Guo, Yu Feng, Yu Wang, Ji Lin, Jingyi Zhang, Shan Wu, Ru Jia, Xiaolei Zhang, Han Sun, Wei Zhang, Wei Li, Hao Hu, Liuyi Jiang

**Affiliations:** ^1^School of Humanities and Social Sciences, Jiangsu University of Science and Technology, Zhenjiang, China; ^2^School of Finance, Zhejiang University of Finance and Economics, Hangzhou, China; ^3^School of Foreign Languages, Nanjing University, Nanjing, China; ^4^Business School, University of Southampton, Southampton, United Kingdom; ^5^School of Sociology and Social Policy, University of Leeds, Leeds, United Kingdom; ^6^Institute of Digital Economy and Green Development, Chifeng University, Chifeng, China; ^7^School of Educational Studies, Mongolian National University of Education, Ulaanbaatar, Mongolia; ^8^Academic Affairs Office, Xing an Vocational and Technical College, Ulanhot, China; ^9^China Center for Economic Research, East China Normal University, Shanghai, China; ^10^School of Economics, Shanghai University, Shanghai, China

**Keywords:** carbon emission trading policy, residents' health, environmental pollution, DID, the moderation effect, China

## Abstract

Residents' health is the basic condition for economic and social development. At present, China's environmental pollution problem is becoming increasingly serious, which not only hinders sustainable economic and social development, but also poses a major threat to public health. Therefore, based on the carbon emissions trading policy implemented in China, this paper explores this policy's impact on residents' health using the DID model and illustrates the moderating effect of environmental pollution. The results show that (1) carbon emissions trading policies can promote the improvement of residents' health; (2) the effect is stronger for western regions and provinces with smaller population sizes after taking control variables into consideration; and (3) environmental pollution has a significant moderating effect on the relationship between carbon emissions trading and residents' health. This research serves as an important reference for expanding the scope of the policy pilot, reducing pollutant emissions, and improving the health of the population.

## Introduction

The theme of the *Healthy China* Strategy is “Joint Construction and Sharing, National Health.” Although China's reform and opening up has achieved rapid economic take-off, it has also brought about serious ecological damage and environmental pollution, and various diseases caused by environmental degradation have seriously hindered the health of the Chinese population. Health is the livelihood issue most directly and closely related to people's real interests. Only by keeping healthy can residents actively participate in production activities and create greater value. Faced with increasingly serious environmental pollution, China has formulated various environmental regulation policies and tried to build a benign and sustainable energy ecosystem by promoting low-carbon production and consumption in pilot provinces, with the aim of achieving the goal of “Healthy China” as quickly as possible. Therefore, exploring the intrinsic relationship between carbon emissions trading policies and residents' health, and studying how pilot policies can improve residents' health by reducing environmental pollution, is beneficial to highlighting the importance of environmental regulation implementation and achieving the goal of “Healthy China.”

So far, the existing literature has focused on the effects of environmental pollution on residents' health and the effects of environmental regulations on infant mortality, laying the foundation for us to explore the effects of environmental regulations on residents' health. However, there are still the following gaps in the literature: First, most studies on residents' health have been conducted through questionnaires to obtain self-assessed health data or have used mortality rates as a proxy for residents' health indices, with the former being more subjective and the latter potentially having biased findings. Second, there are limited studies on the health effects of environmental regulations in China, especially a lack of studies on market-incentivized regulatory policies. In addition, few studies have analyzed their impact paths, leading to a lack of understanding of the impact mechanisms and health benefits of environmental regulations.

Therefore, the innovation of this paper is mainly reflected in the following three aspects: First, the indicator design is innovative. Referring to the WHO's definition of health, five indicators are selected, and the Topsis method of panel data is used to measure the health level of residents, which objectively reflects the health status of residents in 31 Chinese provinces and cities. Second, the research content is expanded and deepened in comparison with previous studies. From the perspective of health improvement and taking market-oriented environmental regulations as an example, this article analyzes the impact of China's carbon emissions trading policy on residents' health and provides references and suggestions for the construction of China's environmental regulation system. Third, this study offers a novel research perspective. Based on an economics perspective, this article combines front-line knowledge and methods of sociology and environmental science to analyze the impact of the interaction between environmental pollution and environmental regulations on residents' health within the context of and literature on regulatory effects, which enriches and expands existing research.

## Literature review and research hypothesis

Research on population health can be traced back to 1972, when Grossman introduced the utility function of health into Becker's household production function and constructed a model of the demand for health capital. The introduction of this function formally opened up research on and exploration of human health. Deng et al. (1) found that adult training had a small positive impact on resident autonomy and a negative impact on resident wellness through a REDCap questionnaire. Ahn et al. (2) believed that long working hours and job-related stress can worsen the health status of residents. Raynor et al. (3) found higher incidence of mental health deterioration in people who were exposed to a COVID-19 shock. Aeschbach et al. (4) selected an active control group for a longitudinal randomized controlled trial and concluded that a tailored MBP may improve certain aspects of resident physicians' positive mental health. Shepley et al. (5) asserted that environmental quality and characteristics can impact both the mental and behavioral health of psychiatric patients. Ke and Chen (6) based on the Health Belief Model, found that residents are living healthier lifestyles after the COVID-19 pandemic in Wuhan, China. Fitzpatrick et al. (7) showed that aboriginal health and mental health problems were more severe in the wake of 2016 Horse River Wildfire in Northern Alberta, Canada. Li et al. (8) claimed that participating in physical exercise can significantly improve the subjective and objective health level of individuals.

Since the industrial revolution, with the progress of society and the development of science and technology, the level of industrialization has been increasing and humanity's ability to exploit natural resources has been rising. As a result, humans have acquired great wealth, but this has come at the price of destroying the natural ecological environment. The decline in environmental quality has caused deterioration of the human living environment, which not only reduces human immunity, but also increases the prevalence of respiratory diseases and the rate of babies suffering congenital defects or health issues, which has a negative impact on human health. For example, the Great Smog of 1952 in London, Photochemical Smog Episode in Los Angeles from 1940 to 1960, Minamata disease incident in Japan in 1956, and Itai-itai Disease in Japan in 1931 have all had demonstrable negative impacts on human health.

Many studies have confirmed that environmental pollution reduces the health status of the population. Qiu et al. (9) found that short-term exposure to elevated concentrations of PM2.5 and NO_2_ were significantly associated with an increased risk of hospital admissions for psychiatric disorders among the US Medicare population. Mallongi et al. (10) believed that air pollution not only has a direct impact on human health, but also can damage the environment. Reginold Raja and Antony Selvi (11) argued that environmental pollution, especially land, air, and water pollution, can harm people's health and exacerbate social problems related to environmental pollution. Sekmoudi et al. (12) showed that long-term exposure to exceedance levels of particulate matter elevated humans' morbidity and mortality. Nwani et al. (13) found that environmental pollution as proxied by per capita CO_2_ emissions has a negative and significant effect on health outcomes in Nigeria. Orru (14) mostly focused on mortality, calculated the health effects of future ozone and particulate matter concentrations under various climate scenarios.

In addition to direct effects, some scholars believe that there are also spillover effects of environmental pollution on residents' health. The First Law of Geography states that correlations between geographic phenomena or attributes are common, and correlations are related to geographic distance; in general, the closer the distance, the greater the correlation, and the farther the distance, the greater the variability Tobler (15). Chen et al. (16) suggested that there is a spatial spillover effect of air pollution on public health, i.e., the health of residents in a certain area is not only affected by local air pollution, but also by air pollution in neighboring areas. This spillover effect of pollution on health was verified in studies by Wang (17), Feng et al. (18), and Song and Cui (19).

To reduce the impact of environmental pollution on a population's health, some countries have formulated environmental regulations guided by the externality theory and attempted to limit pollutant emissions through administrative orders. Chay and Greenstone (20) found that the US *Clean Air Act* implemented in 1970 reduced total suspended particulate concentrations and thereby effectively reduced infant mortality; Luechinger (21) found that the *German Desulfurization Policy* significantly reduced SO_2_ concentrations and infant mortality, and Greenstone and Hanna (22) found that India's *Catalytic Converter Policy* significantly reduced SO_2_ and NO_2_ concentrations and thus contributed to a small reduction in infant mortality. McGartland et al. (23) estimated the health benefits of environmental regulations and concluded that reductions in various environmental pollutants can have a beneficial effect on non-cancer health. Tang et al. (24) came to the conclusion that environmental regulations have a significant co-benefit on high-quality environmental development and public health. However, some studies have also concluded that the policy effects of environmental regulation are limited. Zheng et al. (25) found that China's *Air Pollution Control Law* implemented in 2013 has reduced the population formaldehyde PM2.5 concentration, but the mortality rate showed a non-linear corresponding trend.

Based on the above analysis, this paper proposes the following hypothesis: environmental regulation can promote the improvement of residents' health. Environmental pollution has a positive moderating effect on the relationship between environmental regulation and residents' health.

## Data sources, variables, and models

### Data sources

Considering data availability, completeness, and accuracy, this paper conducts research and analysis based on data from 31 provinces and cities in China from 2009 to 2020. The relevant data are obtained from the China Health Database and the statistical yearbooks of each province and city. For some missing values, the linear interpolation method is used to complete the data.

### Variables' selection

#### Explained variable

The explained variable in this study is resident health (RH). The sum of the individual health of all residents in a country or region is the health status of residents, which is called public health or resident health. The health of residents not only directly affects the happiness of individuals and families, but also affects the social and economic development of the country and/or area. In this paper, the occurrence of diseases among the population is used as a proxy variable for the population's health: the higher the occurrence, the lower the population's health level. Based on the World Health Organization's definition of human health, this paper constructs indicators for measuring the health production function of residents according to the health production function model, and uses the Topsis method of panel data to construct an indicator that can comprehensively measure resident health levels and any changes across different provinces and cities. In this paper, five indicators, namely number of health technicians, number of beds in medical and health institutions, pertussis infection rates, mortality, and average number of times a resident visits a hospital in each region over the study years are used to synthesize the indicator of disease occurrence. Here, the missing values are inferred by linear interpolation.

#### Explanatory variables

This study set environmental regulation (DID) as the explanatory variable. Effective environmental regulations can not only improve the ecological environment, but also have an impact on economic growth and industrial structure, which in turn will further affect residents' health. At present, there is no unified environmental regulation index in academic circles. Some scholars have constructed an index system to synthesize a comprehensive index to characterize the intensity of environmental regulation, and some scholars have used environmental regulation policies implemented by a state/country as indicators of environmental regulation. In this paper, we adopt China's state-implemented carbon emission trading pilot policy as a proxy indicator of environmental regulation to judge the impact of environmental regulation policy on the health level of residents.

#### Moderating variables

This study sets environmental pollution (EP) as the moderating variable. Air pollution is an important risk factor for the health of the population. Studies have demonstrated that reductions in air pollution can reduce infant mortality Chay and Greenstone (20) and improvement of air quality will reduce the mortality rate of the elderly Cesur et al. (26), an effect primarily driven by reductions in cardio-respiratory deaths. The existing literature mostly uses a single indicator to measure environmental pollution, such as the integrated air pollution index Zhang et al. (27) and PM2.5 Bishop et al. (28). However, environmental pollution not only refers to air, but also involves water, soil, and other aspects, so it is one-sided to measure environmental pollution with a single indicator. In this paper, five indicators, namely chemical oxygen demand, ammonia nitrogen emission, sulfur dioxide emissions, nitrogen oxide emissions, and soot emissions in each region over the study years, are used to synthesize an indicator that can comprehensively represent the environmental pollution status through the Topsis method of panel data. Here, the missing values are inferred by linear interpolation.

#### Control variables

In addition to the explanatory variables, some extraneous factors have been identified as possibly also affecting the explained variables. If the influence of these potential factors is ignored, the regression results may become inaccurate. Therefore, five control variables were selected in this paper: urbanization rate (URB), which is measured by the proportion of the resident population in the region's urban area to the total resident population; aging of the population (POP), which is measured by the proportion of the population over 65 years old to the total population; health technicians (HTE), which is measured by the number of health technicians per 1,000 people; average number of resident visits (ANR), which is measured by the ratio of the total number of visits to a hospital at the end of the year to the total resident population; and bed utilization rate (BED), which is measured by the ratio of the actual number of hospital beds used to the actual number of beds available during the period. The descriptive statistics of the variables involved in this paper are shown in Table 1.

**Table 1:** Descriptive statistics of variables.

**VAR**	**Obs**	**Mean**	**Std. Dev**.	**Min**	**Max**
ANR	372	5.210	1.842	1.820	11.650
HTE	372	5.987	1.827	2.365	15.460
BED	372	83.779	7.777	48.300	100.000
URB	372	56.020	13.668	22.700	89.600
POP	372	10.127	2.458	1.000	17.600
RH	372	0.413	0.110	0.177	0.732
EP	372	0.260	0.174	0.003	0.820

### Model selection

#### Model for topsis

Topsis is a method of calculating a composite score for different provinces and municipalities by objectively assigning weights to indicators from three-dimensional data containing years, provinces, cities, and indicators. The indicators need to be selected before the measurement can be performed. Here, we assume that there are 12 years and 31 provinces and cities.

The Topsis measurement process is as follows.

First, the data need to be normalized:


(1) $$\begin{array}{l} \text{Positive var}:\;Std_{ij}=\frac{x_{ij}-min\left\{x_{j}\right\}}{max\left\{x_{j}\right\}-min\left\{x_{j}\right\}} \end{array}$$

(2) $$\begin{array}{l} \text{Negativevar}:\;Std_{ij}=\frac{max\left\{x_{j}\right\}-x_{ij}}{max\left\{x_{j}\right\}-min\left\{x_{j}\right\}} \end{array}$$

After data normalization, the weights of variable *j* in year *i* are calculated and denoted as ω_*ij*_, where *n* represents the observed value:


(3) $$\begin{array}{l} \omega_{ij}=\frac{Std_{ij}}{\sum_{i=1}^{n}Std_{ij}} \end{array}$$

After calculating the weights, the information entropy (*e*_*j*_) and redundancy (*d*_*j*_) of the index are calculated, where *t* represents the year:


(4) $$\begin{array}{l} e_{j}=-\frac{\sum_{i=1}^{n}\omega_{ij}\;*\;ln\omega_{ij}}{lnt} \end{array}$$

(5) $$\begin{array}{l} d_{j}=1-e_{j} \end{array}$$

Then, the weights of the indicators, denoted as ω_*j*_, are calculated:


(6) $$\begin{array}{l} \omega_{j}=\frac{d_{j}}{\sum_{j=1}^{m}d_{j}} \end{array}$$

Finally, the composite index, denoted as *S*_*i*_, is calculated:


(7) $$\begin{array}{l} S_{i}=\overset{m}{\underset{j}{\sum }}\omega_{ij}\;*\;\omega_{j} \end{array}$$

#### Model for DID

Based on the batched pilot time of China's carbon emissions trading policy, this paper constructs three-period panel data, with the seven provinces and cities that have implemented the carbon emissions trading policy as the experimental group and the remaining provinces and cities as the control group. The pilot list contains four municipalities, namely Beijing, Tianjin, Shanghai, and Chongqing, and three provinces, namely Hubei, Guangdong, and Fujian. The difference-in-differences model is as follows:


(8) $$\begin{array}{l} H_{it}=\alpha_{0}+\alpha_{1}DID+\alpha_{2}X_{it}+\lambda_{t}+\mu_{i}+\varepsilon_{it} \end{array}$$

Where *H*_*it*_ represents the health of the population, *DID* is a dummy variable for the carbon trading policy and takes the value of 1 for the year in which the carbon trading policy was implemented and thereafter, otherwise it takes the value of 0. *X*_*it*_ represents the control variable. λ_*t*_ and μ_*i*_ represent the time fixed and provincial and municipal fixed effects, respectively, and ε_*it*_ represents the random error term.

#### Model for parallel trend test

As a policy assessment method, the validity of the DID method is based on the parallel trend test, which means that the experimental group and the control group have a common trend of change before the implementation of the carbon emission trading policy, and the trend of change after the policy's implementation exhibits a difference. In this paper, based on the DID model, we use the dynamic effect coefficient method to conduct the parallel trend test, and the regression model is as follows:


(9) $$\begin{array}{l} H_{it}=\alpha_{0}+\alpha_{k}\overset{t=6}{\underset{t=-4}{\sum }}{DID_{it}}^{t}+\alpha_{2}X_{it}+\lambda_{t}+\mu_{i}+\varepsilon_{it} \end{array}$$

Where ${DID_{it}}^{t}$ represents the year *t* of the implementation of carbon emission trading policy in province or city *i*. The remaining variables are explained in the same way as for model (7). The sample observation period of this paper is 2009–2020, covering 4 years before the implementation of the policy and 6 years after the implementation of the policy in some provinces and cities. In addition, to avoid the effect of cointegration, the year of policy implementation is taken as the base year in this paper and excluded from the regression.

Here, we focus on the confidence interval of the regression coefficient α_*k*_ at the 95% confidence level, which reflects the policy's impact on residents' health before and after the implementation of the carbon emissions trading pilot policy. If the confidence interval includes 0, it indicates that there is no significant difference between the experimental group and the control group. If the confidence interval does not include 0, it is considered that there is a significant difference between the experimental group and the control group. If both sides of the confidence interval are < 0, it indicates that the implementation of the policy has a negative effect on the occurrence of diseases and a positive effect on the health of the population, and if both ends of the confidence interval are >0, it indicates that the implementation of the policy has a negative effect on the occurrence of diseases and a positive effect on the health of the population.

## Empirical result

### Result of topsis

#### Result of the occurrence of disease

The Topsis method with panel data was used to calculate the scores of the occurrence of disease in 31 provinces and cities in China from 2009 to 2020, and Table 2 shows the mean values. According to the calculated results, Xinjiang residents have the highest average occurrence of disease score of 0.623; Beijing comes second with an average value of 0.567, and Qinghai comes in third place with an average value of 0.513. The average score of Jiangsu is the lowest, only 0.286, which is < 50% of Xinjiang's occurrence of diseases. Overall, the occurrence of diseases in 31 provinces and cities in China are mostly in the range of 0.35–0.46, indicating that the level of residents' health still has much room for improvement.

**Table 2:** Average health value of the population in each city and province.

**Province**	**Mean**	**Province**	**Mean**	**Province**	**Mean**
Xinjiang	0.623	Shanghai	0.436	Guangdong	0.369
Beijing	0.567	Inner Mongolia	0.425	Zhejiang	0.368
Qinghai	0.513	Liaoning	0.424	Anhui	0.364
Tibet	0.502	Yunnan	0.414	Chongqing	0.364
Ningxia	0.500	Sichuan	0.406	Hebei	0.354
Heilongjiang	0.485	Guizhou	0.404	Tianjin	0.345
Gansu	0.462	Hainan	0.401	Jiangxi	0.343
Jilin	0.458	Guangxi	0.373	Henan	0.339
Shanxi	0.454	Hunan	0.373	Shandong	0.327
Shaanxi	0.446	Hubei	0.369	Fujian	0.302
Jiangsu	0.286				

In terms of specific values, the occurrence of diseases in all provinces and cities gradually improved over time. During the observation period, Hebei, Hunan, and Jiangsu ranked the top three in terms of the average annual growth rate of disease incidence, with growth rates of 8.11, 7.84, and 7.61%, respectively. Shanghai, Ningxia, and Beijing had the flattest average annual growth rates, with 2.89, 2.86, and 0.83%, respectively. These findings indicate that the medical conditions and health care levels in the provinces and cities improved during the observation period, and therefore, the populations' health levels have improved.

#### Result for environmental pollution

The environmental pollution levels of 31 provinces and cities in China were calculated by the Topsis method from 2009 to 2020, and their average values are shown in Table 3. According to the calculated results, five provinces have environmental pollution levels over 0.4: Jiangsu ranks first with a score of 0.575, Hubei ranks second with a score of 0.535, Anhui ranks third with a score of 0.472, and Hunan and Shandong have scores of 0.452 and 0.433, respectively. This indicates that the above five provinces have higher environmental pollution levels and greater pressure to reduce carbon emissions. Six provinces and cities have environmental pollution scores below 0.1, namely Beijing, Jilin, Ningxia, Shanxi, Guangxi, and Yunnan. Among them, Yunnan has the lowest level of environmental pollution, only 0.013. This indicates that the environmental quality of these six provinces and cities is relatively high.

**Table 3:** Average environmental pollution in each city and province.

**Province**	**Mean**	**Province**	**Mean**	**Province**	**Mean**
Jiangsu	0.575	Xinjiang	0.310	Guizhou	0.196
Hubei	0.535	Hainan	0.301	Inner Mongolia	0.193
Anhui	0.472	Shanghai	0.291	Sichuan	0.159
Hunan	0.452	Jiangxi	0.264	Gansu	0.157
Shandong	0.433	Tibet	0.262	Heilongjiang	0.109
Shaanxi	0.386	Guangdong	0.259	Beijing	0.099
Hebei	0.381	Henan	0.251	Jilin	0.075
Zhejiang	0.380	Qinghai	0.243	Ningxia	0.052
Liaoning	0.352	Fujian	0.241	Shanxi	0.043
Chongqing	0.337	Tianjin	0.217	Guangxi	0.033
Yunnan	0.013				

In terms of specific values, environmental pollution has worsened over time in some provinces, such as Yunnan, Tibet, and Guangxi. The environmental pollution of other provinces and cities has improved, and the average annual growth rate in environmental pollution is negative. The remaining provinces and cities have improved their environmental pollution conditions with negative average annual growth rates for pollution. Beijing, Shanxi, and Jilin had steady average annual growth rates of −16.3, −13.6, and −11.1%, respectively, whereas Jiangxi had an average annual pollution growth rate of only −0.4%. Despite the increasingly severe environmental pollution in some provinces and cities, China has seen a significant improvement overall in environmental quality and a significant decrease in pollutant emission levels.

### Results of DID

In this paper, we use the difference-in-differences method to determine the carbon trading policy's effect on the population's health level. Table 4 shows the regression results of the impact of the carbon emissions trading policy on residents' health. (1) is a DID model without control variables, controlling only for area and time effects, and (2)–(6) are the regression results of gradually including control variables on the basis of (1).

**Table 4:** DID regression results.

**VAR**	**RH(1)**	**RH(2)**	**RH(3)**	**RH(4)**	**RH(5)**	**RH(6)**
DID	−0.058*** (0.0158)	−0.037*** (0.0107)	−0.037*** (0.0109)	−0.033*** (0.0099)	−0.029*** (0.0086)	−0.032*** (0.0087)
URB		0.007*** (0.0016)	0.007*** (0.0016)	0.007*** (0.0014)	0.002 (0.0017)	0.002 (0.0017)
POP			−0.002 (0.0024)	−0.002 (0.0023)	−0.002 (0.0024)	−0.002 (0.0023)
ANR				−0.016*** (0.0056)	−0.014*** (0.0042)	−0.014*** (0.0041)
HTE					0.021*** (0.0027)	0.021*** (0.0025)
BED						−0.002* (0.0009)
Con	0.421*** (0.0022)	0.015 (0.0878)	0.045 (0.0937)	0.142* (0.0798)	0.285*** (0.0903)	0.417*** (0.124)
Obs	372	372	372	372	372	372
*R* ^2^	0.936	0.947	0.947	0.950	0.960	0.961

***, **, and * represent statistical significance at the 1, 5, and 10% levels, respectively. The values in parentheses are robust standard errors for clustering to the province level.

According to Table 4, the estimated coefficients of the carbon emissions trading policy are negative with or without the inclusion of control variables, and they pass the 1% significance test. Thus, it can be concluded that a significant decrease in the occurrence of diseases occurred, which means a significant increase in residents' health levels in the pilot provinces and cities with carbon emissions trading compared with non-pilot provinces after the implementation of the policy. That is, the implementation of the carbon emission trading pilot policy helps to improve the population's health. After gradually adding control variables, the coefficient of the cross-term decreases, indicating that the net effect of the policy decreases, suggesting that control variables such as the average number of residents' medical visits affect the health status of residents to some extent. However, in general, the carbon trading policy will promote the improvement of the population's health, which confirms the first hypothesis.

Regarding the regression results of the control variables, the increase of the control variables raises the decidable coefficient *R*^2^, indicating that the control variables were effectively selected. Among them, the regression coefficients of urbanization are mostly significantly positive, indicating that the occurrence of diseases rises with the level of urbanization. The coefficient of the proportion of the population over 65 years old does not pass the significance test. The coefficient of average number of resident visits is significantly negative at the 1% level, which indicates that higher the number of resident visits, the worse the residents' health status. The coefficient of bed utilization rate is significantly negative at the 10% level, indicating that the higher the bed utilization rate, the lower the occurrence of disease.

### Robustness tests

#### Parallel trend test

**Figure 1:**
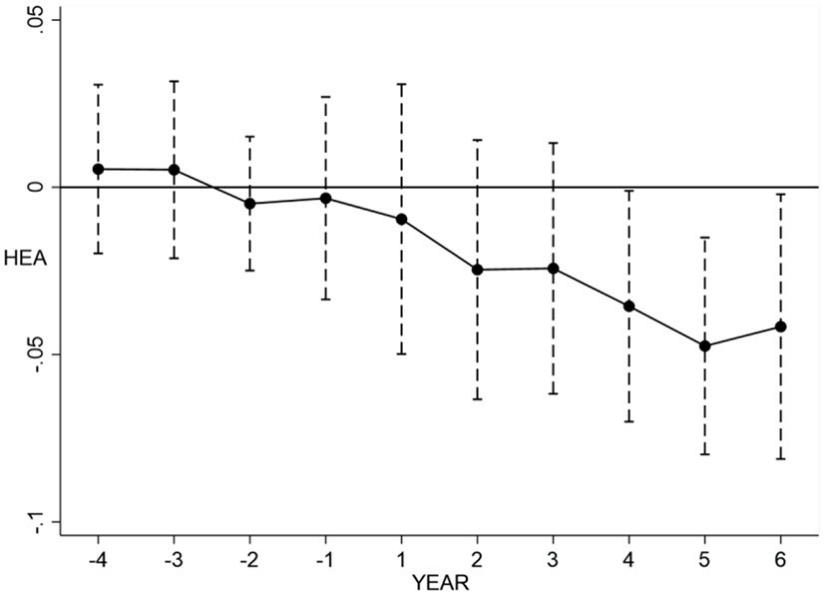
Parallel trend test graph.

In order to visually examine the impact of China's carbon trading policy on residents' health, this paper plots the dynamic effect coefficients of the impact of this policy on residents' health. Figure 1 depicts the regression coefficients and 95% confidence intervals of the regression coefficient α_*k*_. According to Figure 1, no significant difference existed between the occurrence of diseases in the experimental group and control group before implement of the carbon emissions trading policy. In the first 3 years after the policy was implemented, the difference remained insignificant, but the confidence interval does not contain 0 from the fourth year, indicating that the data used in this paper satisfy the parallel trend hypothesis and there is a 3 year lag in the effect of the carbon emissions trading on residents' health. This verifies the robustness of previous study findings. The existence of such a policy lag may be attributable to the fact that the carbon emissions trading system needs to go through approval, construction, and operation to take full effect; this process will take a certain amount of time. Only after a certain amount of time has accumulated can the policy effect be given full play.

#### PSM-DID test

Although the DID method can analyze the net effect of the policy and solve some endogeneity problems, sample selection bias is likely to exist because the pilot regions for implementing the carbon emission trading policy were not randomly determined, and the initial conditions of pilot provinces and cities are significantly different from those of non-pilot provinces and cities. To avoid endogeneity problem caused by sample selection bias, this paper further adopts the PSM-DID model to re-estimate the matched sample values by eliminating observations that do not satisfy the common area assumption based on the nearest-neighbor matching.

According to the results of the equilibrium test for continuous variables, the absolute value of the standard deviation of the control variables after matching is < 20%, indicating that the matching is effective; and the probability *P* of the *T*-value is much >10%, indicating that the propensity score matching is more effective. Table 5 reports the results of the DID model estimation for the carbon emissions trading policy after sample matching. According to Table 5, the results after controlling for year effects and provincial and municipal effects are generally consistent with the results of the main regression. The results show that the carbon emissions trading policy has a negative effect on occurrence of diseases; that is, the effect on residents' health is significantly positive, which is basically consistent with the results estimated by the DID method. Thus, the robustness of the main regression analysis in this paper, i.e., China's carbon trading policy has contributed to the improvement of the residents' health, is proven.

**Table 5:** PSM-DID regression results.

**VAR**	**RH**	**RH**
DID	−0.016* (0.0092)	−0.030*** (0.0086)
Cons	0.299*** (0.0081)	0.451*** (0.1282)
Time*Id	Yes	Yes
Control variables	No	Yes
Obs	177	177
*R* ^2^	0.887	0.914

***, **, and * represent statistical significance at the 1, 5, and 10% levels, respectively. The values in parentheses are robust standard errors for clustering to the province level.

#### Tailoring test

To avoid the influence of outliers on the regression results, this paper applies the upper and lower 5% tailing treatment to all continuous variables, and then re-runs the regression. The re-run regression results are shown in Table 6. It is not difficult to find that the regression coefficient of carbon trading policy is still significantly negative after the tailing treatment, which indicates that the implementation of carbon trading policy led to reduced occurrence of diseases, showing that the policy can contribute to the improvement of the health of the population, which is consistent with the findings of the above study.

**Table 6:** Results of tailoring regression.

**VAR**	**RH**	**RH**
DID	−0.050*** (0.0167)	−0.024** (0.0093)
Cons	0.418*** (0.0023)	0.480*** (0.0989)
Time*Id	Yes	Yes
Control variables	No	Yes
*R* ^2^	0.915	0.944

***, **, and * represent statistical significance at the 1, 5, and 10% levels, respectively. The values in parentheses are robust standard errors for clustering to the province level.

## Test for heterogeneity and moderating effect

### Results of heterogeneity analysis

#### Regional heterogeneity

The above analysis has shown that China's carbon trading policy has a significant promoting effect on residents' health. This finding gives rise to other questions, such as: Are the health effects still present in different areas? If so, are there significant differences between regions? To further study the regional heterogeneity of the pilot policy, this paper further divides the research sample into three regions, namely Eastern, Central, and Western, and explore the differences of impacts of the carbon emissions trading policy on residents' health in different regions from the perspective of regional heterogeneity. The results are shown in Table 7.

**Table 7:** Results of regional heterogeneity.

**VAR**	**Eastern**	**Eastern**	**Central**	**Central**	**Western**	**Western**
DID	−0.056*** (0.0161)	−0.028* (0.0148)	−0.012 (0.0148)	−0.033*** (0.0072)	−0.034*** (0.0091)	−0.046*** (0.0119)
Cons	0.396*** (0.0045)	0.433 (0.2901)	0.399*** (0.0005)	0.569*** (0.1910)	0.465*** (0.0005)	0.475** (0.2253)
Time*Id	Yes	Yes	Yes	Yes	Yes	Yes
Control variables	No	Yes	No	Yes	No	Yes
*R* ^2^	0.930	0.958	0.942	0.962	0.961	0.972

***, **, and * represent statistical significance at the 1, 5, and 10% levels, respectively. The values in parentheses are robust standard errors for clustering to the province level.

The results show that without considering the control variables, the carbon emissions trading policy's effect on the occurrence of diseases is the largest in the east, second largest in the west, and the smallest in the central region. Thus, the carbon emissions trading policy has differing effects on the occurrence of diseases in different regions. Its effect on the health of residents in the east and west is significantly negative, while not significant in the central region. After considering urbanization level and other control variables, the policy's effect on residents' health is the largest in the west, second largest in the central region, and the smallest in the central region. The policy's effect on residents' health in the central and western regions is the most significant, and the effect in the eastern region is relatively weak. This is probably because the economic development level of the central and western regions is slightly lower than that of the eastern region. The central and western regions are in the primary stage of industrial structure transformation, and the scale of pollutant emissions in that process of industrial development is larger. Therefore, the carbon trading policy has a greater emission reduction effect and a stronger health enhancement effect on populations in those regions. In addition, most of the production sectors in the eastern region have been transferred to the central and western regions, and now the eastern region mainly focuses on the development of high-tech manufacturing and multifunctional manufacturing centers, so the effect of carbon emissions trading on the health of residents is limited.

#### Scale heterogeneity

Compared with provinces with large populations, provinces with smaller populations have less human capital, a relatively weaker industrial base, and higher health expenditure per capita, which may lead to differences in carbon emission trading pilot policies among provinces with different population sizes. To assess any such impact, this paper further divides the research samples into large and small scales according to the total population. Large provinces are defined as those with a total population exceeding 50 million; otherwise, they are considered small provinces or cities. Then, from the perspective of population size heterogeneity, this paper explores the differences in the carbon emissions trading policy's impact on the health of residents in provinces of different sizes. The results are shown in Table 8.

**Table 8:** Results of scale heterogeneity.

**VAR**	**Large**	**Large**	**Small**	**Small**
DID	−0.026* (0.0136)	−0.135 (0.1300)	−0.071*** (0.0190)	−0.038*** (0.0122)
Cons	0.359*** (0.0017)	0.217 (0.2161)	0.450*** (0.0027)	0.449*** (0.1387)
Time*Id	Yes	Yes	Yes	Yes
Control variables	No	Yes	No	Yes
*R* ^2^	0.939	0.958	0.929	0.956

***, **, and * represent statistical significance at the 1, 5, and 10% levels, respectively. The values in parentheses are robust standard errors for clustering to the province level.

The regression results show that significant heterogeneity exists in the impact of carbon trading policies on the population's health across provinces of different sizes. When the control variables are not considered, the coefficient and significance level of the impact of carbon emission trading policy on the occurrence of diseases decrease as the increase of population size in each province. After taking the control variables into consideration, the impact of carbon emissions trading policies on the occurrence of diseases was significantly negative in areas with a smaller population size, and the coefficient of impact increased but was no longer significant as the population size increased. This may be because the expansion of population increases pressure on original medical resources in the area. Additionally, the expansion of population scale brings improvements in infrastructure and confers the advantage of agglomeration, which lays a solid foundation for the development of industrial industry. The development of industry further increases environmental pollution, which has a negative impact on the population's health, and thus environmental regulation has a stronger effect on enhancing the population's health in provinces with a smaller population size.

### Results of the moderation effect

As an environmental regulation policy, China's carbon emissions trading policy can reduce environmental pollution, which in turn affects the occurrence of diseases. Therefore, this paper first measured the environmental pollution of each province and city through the Topsis method, and then calculated the cross term (*TT*) of carbon trading and environmental pollution to investigate the moderating role of environmental pollution in the process of China's carbon trading policy's ability to improve the health level of residents. The regression model was first tested before conducting the main analysis, and the results of Hausman test rejected the random effect hypothesis; thus, the fixed effect model was chosen. The regression results are shown in Table 9.

**Table 9:** Results of moderation effect.

**VAR**	**RH(1)**	**RH(2)**	**RH(3)**
DID	0.026* (0.0154)	−0.016 (0.0271)	0.027* (0.0154)
EP	−0.506*** (0.0378)	−0.520*** (0.0384)	−0.497*** (0.0379)
TT		0.167* (0.0885)	
TT-N			0.167* (0.0885)
Cons	0.541*** (0.0111)	0.545*** (0.0113)	0.539*** (0.0111)
Time*Id	Yes	Yes	Yes
Control variables	No	No	No
*R* ^2^	0.380	0.386	0.386

***, **, and * represent statistical significance at the 1, 5, and 10% levels, respectively. The values in parentheses are robust standard errors for clustering to the province level.

According to the results, the coefficient of the interaction term (*TT*) between environmental regulation and environmental pollution is significantly negative at the 10% level, indicating that environmental pollution is a moderating variable on the relationship between the occurrence of diseases and carbon trading policies. Comparing the regression results, the originally significant *did* was not significant after adding the interaction term, which may be due to the high cointegration between the interaction term and the carbon trading and environmental pollution that biased the model estimation. Therefore, we further correct the sample data for centrality and then rerun the regression, and the results are shown in the third column. The result shows that environmental regulation also becomes significant after decentering, and the new interaction term *TT-N* does not change, suggesting that carbon trading policies can improve the population's health by reducing environmental pollution. This confirms the second hypothesis.

## Conclusion and implications

This paper used the data on 31 provinces and cities in China from 2009 to 2020 to study the impact of carbon emission trading policies on residents' health using the difference-in-differences model. The results show that (1) carbon emissions trading policies can promote the improvement of residents' health; (2) this improvement effect is heterogeneous among provinces and cities in different regions and with population sizes, and the effect is stronger for western regions and provinces with smaller population sizes after taking control variables into consideration; and (3) environmental pollution has a significant moderating effect on the relationship between carbon emissions trading and residents' health.

In view of the above conclusion and the actual situation in China, the following insights are obtained in this study and the following recommendations can be made: First, the scope of policy pilots should be expanded, and environmental regulation policies should be implemented. Research shows that the carbon emissions trading policy has a significant effect on the health of residents. Therefore, to reduce the incidence of diseases and improve the population's health, the scope of carbon emissions trading pilot projects can be expanded and regulation can be strengthened to reduce pollutant emissions and achieve a harmonious coexistence between human health and the natural environment. Second, the actual situation and local conditions should be considered. The impact of China's carbon emissions trading policy on the health of residents in different regions and provinces with different population sizes is heterogeneous. Thus, in the process of policy implementation, each region should consider its local conditions, combine the characteristics of the region's economic level, population size, and resource endowment to explore a low-carbon development path suitable for the region, and formulate a more scientific and reasonable carbon emissions trading policy. Third, publicity should be increased regarding the low-carbon concept and offering guidance for residents to live a low-carbon life. Environmental pollution, as a regulating variable, has a moderating effect on the relationship between residents' health and the carbon emissions trading policy. It is possible to provide low-carbon public goods and reasonable subsidies so that the low-carbon concept can be deeply rooted in people's hearts and thus improve residents' health.

There are still some limitations that could be explored in further research. On the one hand, this paper uses the occurrence of diseases as a proxy for population health rather than directly measuring residents' health levels. On the other hand, due to data limitations, the study in this paper is limited to the provincial level, and as micro-entities of pollutant emission, the role of the carbon emission trading policy on the pollution emissions of enterprises should be noticed. We believe that future work will provide useful supplements in these aspects.

## Data availability statement

The original contributions presented in the study are included in the article/Supplementary material, further inquiries can be directed to the corresponding author/s.

## Author contributions

BG and YF designed the study, performed the research, analyzed data, and wrote the paper. YW and SW collected most of the data. JZ and RJ checked the spelling of the paper and corrected authors the mistakes. JL provided fund support and suggestions response on revising the paper. XZ and HS proposed some countermeasures. WZ and WL verified the authenticity of the data analysis. HH and LJ presented the methodology. All authors contributed to the article and approved the submitted version.

## Funding

This research was supported by National Social Science Fund of China (Grant No. 20BJL040) and Graduate Research and Innovation Projects of Jiangsu Province (Grant No. KYCX22_3727).

## Conflict of interest

The authors declare that the research was conducted in the absence of any commercial or financial relationships that could be construed as a potential conflict of interest.

## Publisher's note

All claims expressed in this article are solely those of the authors and do not necessarily represent those of their affiliated organizations, or those of the publisher, the editors and the reviewers. Any product that may be evaluated in this article, or claim that may be made by its manufacturer, is not guaranteed or endorsed by the publisher.
